# Intranasal Administration of an Arginine‐Enriched Penetratin Peptide Confers Neuroprotection via Mitochondrial Functional Modulation in a Genetic Parkinson's Disease Model

**DOI:** 10.1002/cns.71108

**Published:** 2026-08-26

**Authors:** Jui‐Chih Chang, Cheng‐Yi Yeh, Kai‐Li Liu, Yi‐Chun Chao, Chin‐Hsien Lin, Chin‐San Liu

**Affiliations:** ^1^ Center of Regenerative Medicine and Tissue Repair Institute of ATP, Changhua Christian Hospital Changhua Taiwan; ^2^ Department of Nutrition Chung Shan Medical University Taichung Taiwan; ^3^ Department of Nutrition Chung Shan Medical University Hospital Taichung Taiwan; ^4^ Laboratory Animal Center Changhua Christian Hospital Changhua Taiwan; ^5^ Department of Neurology National Taiwan University Hospital Taipei Taiwan; ^6^ Vascular and Genomic Center Institute of ATP, Changhua Christian Hospital Changhua Taiwan; ^7^ Department of Neurology Changhua Christian Hospital Changhua Taiwan; ^8^ Graduate Institute of Integrated Medicine, College of Chinese Medicine China Medical University Taichung Taiwan

**Keywords:** cell‐penetrating peptide, intranasal delivery, mitochondrial dysfunction, neuroprotection, parkinson's disease, *UQCRC1* mutation

## Abstract

**Background:**

Mitochondrial dysfunction is a central pathogenic mechanism in Parkinson's disease (PD), particularly in genetic forms associated with respiratory chain impairment. Disease‐modifying therapies capable of restoring neuronal bioenergetics while achieving non‐invasive brain delivery remain lacking. This study investigated the therapeutic potential of an arginine‐enriched penetratin‐derived peptide (PenArg) in a genetic PD model carrying the *UQCRC1* (p.Tyr314Ser) mutation.

**Methods:**

The effects of PenArg labeled with 5‐FAM– or biotin were evaluated in *UQCRC1* knock‐in SH‐SY5Y cells and mice. Mitochondrial function, neuronal survival, and apoptosis were assessed using biochemical and imaging analyses. Brain distribution was evaluated 2 h after intranasal administration, and therapeutic efficacy was examined following chronic intranasal treatment (three times weekly for six months).

**Results:**

PenArg restored mitochondrial membrane potential, improved neuronal viability, and reduced oxidative stress‐induced apoptosis in cellular models. Imaging studies demonstrated partial mitochondrial localization in vitro and in vivo. Intranasal administration significantly improved locomotor performance, preserved dopaminergic neurons in the substantia nigra (SN) and striatum, and maintained hippocampal neuronal structure. Brain distribution analysis confirmed widespread brain penetration after intranasal administration. PenArg restored ATP synthase subunit beta expression and complex III activity while reducing cytochrome c release and caspase‐3 activation in SN neurons. Treatment additionally attenuated skeletal muscle atrophy, enhanced antioxidant responses, and reduced circulating pro‐inflammatory cytokines.

**Conclusion:**

PenArg confers neuroprotection through functional modulation of mitochondrial homeostasis and produces multisystem therapeutic benefits. Intranasal administration represents a non‐invasive strategy with disease‐modifying potential for mitochondrial‐related neurodegenerative disorders.

## Graphical abstract

Intranasal PenArg achieved broad brain delivery and improved mitochondrial function in a UQCRC1‐mutant Parkinson's disease model. Chronic treatment preserved dopaminergic neurons, improved motor performance, reduced neuroinflammatory and systemic inflammatory responses, and attenuated skeletal muscle pathology.

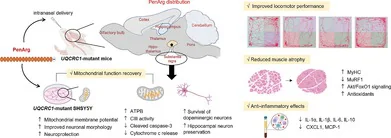

**Abbreviations**
ATPB: ATP synthase subunit beta
BBB: blood–brain barrier
CIII: mitochondrial complex III
CNS: central nervous system
CPP: cell‐penetrating peptide
Cyt c: cytochrome c
DG: dentate gyrus
DG‐GCL: dentate gyrus granule cell layer
HP: hippocampus
MMP: mitochondrial membrane potential
MuRF1: muscle RING‐finger protein‐1
MyHC: myosin heavy chain
PD: Parkinson's disease
PenArg: arginine‐enriched penetratin
SN: substantia nigra
ST: striatum
TA: tibialis anterior
TH: tyrosine hydroxylase
UQCRC1: ubiquinol‐cytochrome c reductase core protein 1

## 1 Introduction

Parkinson's disease (PD) is a progressive neurodegenerative disorder characterized by the selective loss of dopaminergic neurons in the substantia nigra (SN), frequently associated with mitochondrial dysfunction, a central driver of disease progression. *UQCRC1*, a nuclear‐encoded subunit of mitochondrial complex III, is essential for oxidative phosphorylation. A missense mutation in *UQCRC1* (p.Tyr314Ser, p.Y314S) has been identified in familial PD, leading to impaired complex III activity, oxidative stress, and neuroinflammation [1]. *UQCRC1*‐mutant models replicate these pathological features and provide a valuable platform for evaluating mitochondria‐targeted therapeutic strategies [1, 2]. Current dopaminergic therapies provide symptomatic relief but do not address the underlying mitochondrial bioenergetic failure, particularly in genetic PD associated with *UQCRC1* mutation. Strategies that restore mitochondrial function while enabling brain delivery through an intranasal route that bypasses the blood–brain barrier (BBB) represent an unmet therapeutic need [3, 4, 5].

Cell‐penetrating peptides (CPPs) are short amino acid sequences capable of traversing cellular membranes and the BBB, making them promising candidates for neurological therapy [6, 7, 8]. Beyond serving as delivery vectors9, arginine‐rich CPPs such as Arg‐9 (R9) and penetratin exhibit intrinsic cytoprotective effects independent of cargo conjugation [7, 9, 10], including preservation of mitochondrial membrane potential, attenuation of oxidative stress, and suppression of apoptotic signaling [11, 12, 13]. Whether these intrinsic properties translate into disease‐modifying effects in genetic models of mitochondrial dysfunction remains unclear.

A penetratin analog enriched with arginine residues (PenArg) was designed to enhance cellular uptake through strengthened electrostatic interactions with negatively charged membranes [14]. Given its high cationic charge density and potential interaction with mitochondrial membranes [15, 16, 17], PenArg may exert biological effects beyond cargo delivery [11]. However, its therapeutic relevance in mitochondrial‐related neurodegeneration has not been defined.

In this study, we investigated the therapeutic effects of PenArg in vitro and in vivo in a *UQCRC1*‐mutant PD model, assessing mitochondrial function, neuronal survival, brain distribution after intranasal delivery, and long‐term outcomes to determine its potential as a standalone neuroprotective agent.

## 2 Methods

### 2.1 Cell Culture and Peptide Treatment

Human SH‐SY5Y neuroblastoma cells (ATCC, CRL‐2266, Manassas, VA, USA), including wild‐type (WT) and UQCRC1 knock‐in lines, were maintained and used in an undifferentiated state throughout the in vitro experiments. Cells were cultured in DMEM/F12 medium (Gibco, Thermo Fisher Scientific, Waltham, MA, USA) supplemented with 10% fetal bovine serum (FBS; Gibco, Thermo Fisher Scientific, Waltham, MA, USA) and 1% penicillin–streptomycin (Gibco, Thermo Fisher Scientific, Waltham, MA, USA) at 37°C with 5% CO_2_.

PenArg (RQIRIWFQNRRMRWRRC‐NH₂, Kelowna International Scientific Inc., Taipei, Taiwan) was synthesized and purified by high‐performance liquid chromatography (HPLC) to > 98% purity. PenArg was applied at 0.5–20 μM for 24 h. Cytoprotection was assessed after 24 h PenArg pretreatment followed by 10 μM t‐BH (Sigma‐Aldrich, St. Louis, MO, USA) exposure for 18 h. For peptide tracing and localization experiments, 5‐FAM–PA‐Cys was synthesized, with a molecular weight of 2819.25 Da confirmed by mass spectrometry. 5‐FAM–PenArg was incubated for 6 h prior to fixation.

### 2.2 Assessment of Cell Morphology, Mitochondrial Function and Apoptosis

Mitochondrial membrane potential (MMP) was assessed using tetramethylrhodamine ethyl ester (TMRE; Invitrogen, Thermo Fisher Scientific, Waltham, MA, USA) and quantified with a microplate reader (BioTek, Winooski, VT, USA), normalized to Hoechst 33342 staining (Invitrogen, Thermo Fisher Scientific). Mitochondrial depolarization and apoptosis were evaluated using MitoSense Red and 7‐aminoactinomycin D (7‐AAD) with the MMP and Apoptosis Assay Kits (ChemoMetec, Allerod, Denmark) on a NucleoCounter NC‐3000 system (ChemoMetec). Cell morphology and filopodia length were analyzed by phase‐contrast microscopy (Olympus, Tokyo, Japan) and ImageJ (v1.54d, National Institutes of Health, Bethesda, MD, USA).

### 2.3 Cellular Distribution of 5‐FAM‐PenArg

Mutant cells were incubated with 5‐FAM‐PenArg (10 μM, 16 h, 37°C), fixed with 4% paraformaldehyde (PFA; Sigma‐Aldrich, St. Louis, MO, USA), and counterstained with DAPI. Images were acquired using an Olympus FluoView FV1200 confocal microscope (Olympus, Tokyo, Japan). Z‐stack images (21 sections, 0.5 μm intervals) and longitudinal reconstructions were generated using Fluoview Viewer software (Olympus).

### 2.4 Animal Study

All animal procedures were conducted in accordance with AAALAC guidelines and approved by the Animal Experiments and Ethics Committee of Changhua Christian Hospital. *UQCRC1* p.Y314S knock‐in mice were generated by CRISPR/Cas9 genome editing and obtained from Dr. Chin‐Hsien Lin [1]. Mice were backcrossed with C57BL/6J mice (BioLASCO, Taipei, Taiwan) for ≥ 3 generations. Genotyping was performed by PCR using primers VF1 (5′‐CCACGTGGCCATTGCAGTAGA‐3′) and VR1 (5′‐TCGATACTCATGGCATCACAGAC‐3′), followed by BsrGI digestion, as previously described [1]. Heterozygous mice were used for experiments, with WT littermates as controls. Both male and female mice were included, with sex balanced across groups where possible. Eight‐month‐old mice were randomized into four groups: WT, UQCRC1 mutant (UQ), UQCRC1 mutant receiving vehicle treatment (UQ‐Sham), and UQCRC1 mutant treated with PenArg (UQ‐PA) (*n* = 6 per group). Animals were housed under standard conditions with ad libitum access to food and water.

Biotinylated PenArg was synthesized as a cysteine‐conjugated PenArg derivative for intranasal administration. Its molecular weight was confirmed by mass spectrometry as 2559.98 Da, and peptide purity was verified by HPLC to be > 98%. The final concentration of DMSO (Sigma‐Aldrich, St. Louis, MO, USA) in the intranasally administered formulation was approximately 7%. For intranasal administration, awake mice received PBS containing DMSO as vehicle control or biotinylated PenArg (0.5 mM) in a total volume of 20 μL (10 μL per nostril) using QSP 20 μL filter tips (Thermo Fisher Scientific, Waltham, MA, USA), as previously described [18]. Intranasal administration was performed three times per week for six months. The intranasal dose was based on the effective in vitro concentration [19].

### 2.5 Open Field Behavioral Test

Locomotor activity was assessed using an open field system (EthoVision XT, Noldus Information Technology, Wageningen, The Netherlands). Mice were placed in a 40 × 40 cm arena for 10 min, and movement parameters were automatically recorded.

### 2.6 Histological and Immunostaining Analysis

Mouse brains were perfused with PBS and 4% paraformaldehyde (PFA), paraffin‐embedded, and sectioned at 3 μm for histological staining and immunohistochemistry. For immunofluorescence staining, brain sections were prepared at 10 μm thickness. Tyrosine hydroxylase (TH) immunohistochemistry (IHC) was performed following citrate antigen retrieval (10 mM sodium citrate, pH 6.0; Sigma‐Aldrich, St. Louis, MO, USA) using anti‐TH (1:200; Novus Biologicals, Littleton, CO, USA) and HRP‐conjugated secondary antibody (1:200; Millipore, Billerica, MA, USA), with 3,3′‐diaminobenzidine (DAB; DakoCytomation, Carpinteria, CA, USA) visualization. Nissl staining was conducted with cresyl violet (Sigma‐Aldrich). To assess neuroinflammatory activation in the SN, immunofluorescence staining was performed using anti‐Iba‐1 antibody, a microglial marker, ionized calcium‐binding adapter molecule 1 (1:100; Bioss Inc., Woburn, MA, USA), and anti‐GFAP antibody, glial fibrillary acidic protein (1:200; GeneTex Inc., Irvine, CA, USA). Staining intensity in SN, striatum (ST), and dentate gyrus (DG), as well as Iba‐1 and GFAP fluorescence intensity in the SN, was quantified using ImageJ (v1.54d, National Institutes of Health, Bethesda, MD, USA).

Biotinylated PenArg was detected using streptavidin–HRP (1:200; Abcam, Cambridge, MA, USA) on sagittal sections (2 h post‐administration). Mitochondrial localization (6‐month endpoint) was assessed by co‐immunohistochemistry with anti‐ATPB (1:1000; Abcam).

### 2.7 Western Blotting

Substantia nigra (SN) and tibialis anterior (TA) tissues were homogenized in RIPA buffer (Thermo Fisher Scientific, Waltham, MA, USA) with protease and phosphatase inhibitors (Roche Diagnostics, Taipei, Taiwan). Protein concentration was determined by BCA assay (Thermo Fisher Scientific), and equal amounts (20–30 μg) were subjected to SDS‐PAGE and transferred to PVDF membranes (Millipore, Billerica, MA, USA). Primary antibodies included cleaved caspase‐3 (1:1000; Cell Signaling Technology, Danvers, MA, USA), cytochrome c (1:1000; Abcam, Cambridge, MA, USA), ATPB (1:1000; Abcam), MuRF1 (1:1000; ECM Biosciences, Aurora, CO, USA), MyHC (1:1000; Thermo Fisher Scientific), Akt, p‐Akt, p‐FoxO1, p‐FoxO3a (1:1000; Cell Signaling Technology), catalase and SOD2 (1:1000; GeneTex, Hsinchu City, Taiwan), and GAPDH (1:2000; Millipore). Signals were detected using enhanced chemiluminescence (ECL; PerkinElmer Life Sciences, Boston, MA, USA), visualized with LAS‐1000 Plus (Fuji Photo Film Co., Tokyo, Japan), and quantified using AlphaEase FC software (Alpha Innotech Corp., San Leandro, CA, USA).

### 2.8 Measurement of Mitochondrial Complex III Activity

Mitochondrial complex III (CIII) activity in SN lysates was measured using a commercial assay kit (BioVision, Milpitas, CA, USA) and normalized to total protein content.

### 2.9 Histology and Morphometric Analysis of Skeletal Muscle

TA muscle sections were stained with hematoxylin and eosin (H&E) to assess fiber morphology. Cross‐sectional area and fiber density were quantified using ImageJ from at least three random fields per section.

### 2.10 Cytokine Analysis

Blood was collected by cardiac puncture into EDTA Vacutainer tubes (Becton, Dickinson and Co., Franklin Lakes, NJ, USA). Plasma was isolated, supplemented with protease inhibitor cocktail (1:100; Millipore, St. Charles, MO, USA), and stored at −80°C. Cytokines (IFNγ, TNF‐α, IL‐1α, IL‐1β, IL‐6, IL‐10, IL‐12 (p70), CXCL1/KC, MCP‐1/CCL2) were quantified using a multiplex bead‐based immunoassay (MAGPIX, Millipore, St. Charles, MO, USA) and analyzed with MILLIPLEX Analyst software (v5.1; VigeneTech, Carlisle, MA, USA).

### 2.11 Statistical Analysis

Data are presented as mean ± SD (cell experiments) or mean ± SEM (animal experiments). Statistical analysis was performed using one‐way ANOVA with Tukey's post hoc test or a two‐tailed unpaired Student's *t*‐test (GraphPad Prism 10, GraphPad Software, San Diego, CA, USA), with *p* < 0.05 considered significant.

## 3 Results

### 3.1 Mitochondrial Localization and Neuroprotective Effects of PenArg in Vitro

**FIGURE 1:**
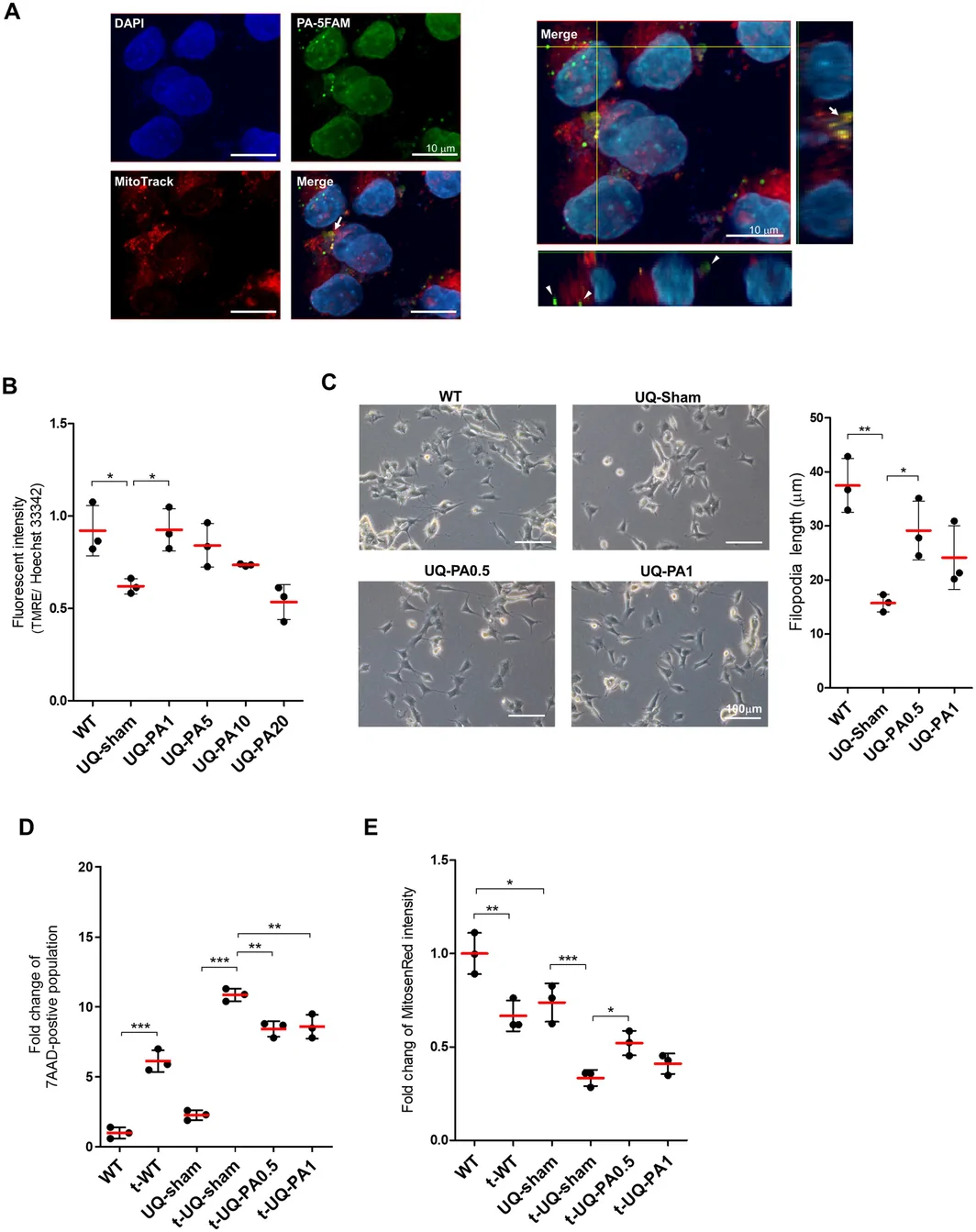
Cellular uptake of PenArg and its effects on mitochondrial membrane potential, neuronal morphology, and apoptosis in *UQCRC1* knock‐in SH‐SY5Y cells. (A) Confocal microscopy showing intracellular uptake and distribution of 5‐FAM‐labeled PenArg (10 μM, 16 h). Nuclei were counterstained with DAPI. Z‐stack reconstruction and longitudinal views generated from integrated z‐axis line scans confirmed intracellular localization of the peptide. (B) Quantification of mitochondrial membrane potential (MMP) using TMRE fluorescence in wild‐type (WT) and *UQCRC1* knock‐in SH‐SY5Y cells (UQ) treated with various doses of PenArg (1, 5, 10, 20 μM) for 24 h. (C) Representative phase‐contrast images showing cell morphology and filopodium formation after 24‐h treatment with 0.5 μM and 1 μM PenArg. Right panel: Quantification of average filopodia length. (D) Quantification of apoptotic cells using 7‐AAD staining after tert‐butyl hydroperoxide (t‐BH) treatment for 16 h in WT and UQ cells, with or without peptide pretreatment. (E) Fold change of MitoSense Red fluorescence intensity indicating MMP under oxidative stress. All data are shown as mean ± SD; *p < 0.05, **p < 0.01, ***p < 0.001, *n* = 3 per group.

The *UQCRC1*‐mutant SH‐SY5Y cells treated with 10 μM 5‐FAM–PenArg for 6 h showed predominant nuclear localization with partial mitochondrial colocalization (Figure 1A, arrows). Z‐stack reconstruction confirmed mitochondrial localization (Figure 1A, right panel, arrows), with limited cytosolic diffusion (arrowheads). TMRE analysis demonstrated reduced baseline MMP in mutant cells compared with WT controls. Low‐dose PenArg (1 μM) increased TMRE intensity, whereas higher concentrations attenuated this effect (Figure 1B). Subsequent experiments therefore focused on 0.5–1 μM. At 0.5 μM, PenArg enhanced filopodia formation (Figure 1C). Although *UQCRC1*‐mutant cells showed reduced TMRE intensity and impaired filopodia formation under basal conditions, the percentage of 7‐AAD‐positive cells was not significantly increased compared with WT cells. Under oxidative stress challenge, PenArg reduced apoptosis, as indicated by decreased 7‐AAD positivity (Figure 1D), and preserved MMP under t‐BH exposure (Figure 1E). Overall, 0.5 μM PenArg most effectively preserved mitochondrial integrity and reduced stress‐induced apoptosis.

### 3.2 Improvement of Locomotor Function by PenArg Treatment in *UQCRC1* ‐Mutant Mice

**FIGURE 2:**
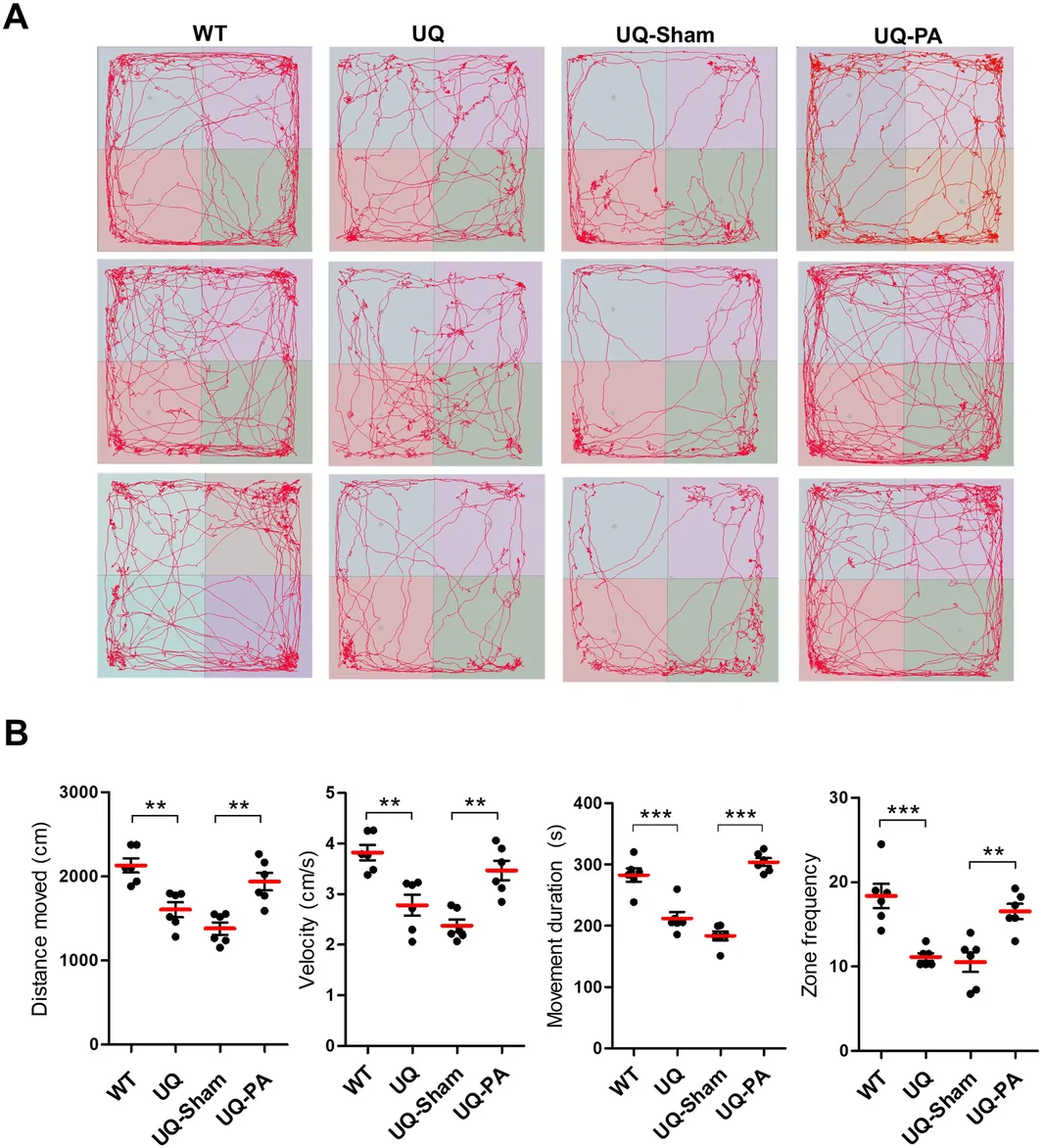
Improvement of locomotor activity in treated *UQCRC1*‐mutant mice. (A) Representative locomotor trajectories from open field testing were shown for Wild‐type (WT), *UQCRC1*‐mutant (UQ), vehicle‐treated mutant (UQ‐Sham), and PenArg‐treated mutant (UQ‐PA) mice, with three examples per group. (B) Quantitative analysis of locomotor parameters, including total distance moved (cm), velocity (cm/s), movement duration (s), and zone crossing frequency. All data are presented as mean ± SEM; *p < 0.05, **p < 0.01, ***p < 0.001, *n* = 6 per group.

Compared with WT littermate mice, *UQCRC1*‐mutant mice (UQ), including those receiving vehicle treatment, exhibited marked reductions in locomotor activity, characterized by sparse and fragmented movement patterns (Figure 2A). In contrast, mice treated with PenArg (UQ‐PA) showed recovery in locomotor behavior, with trajectories resembling WT mice. Quantitative analysis of movement parameters revealed significant and consistent improvements in the UQ‐PA group across all measured metrics (Figure 2B), including total distance moved, velocity, movement duration, and number of zone crossings among the four predefined zones (zone crossing frequency).

### 3.3 PenArg Treatment Preserves Nigrostriatal Dopaminergic Neurons, Attenuates Mitochondrial Apoptosis‐Associated Changes, and Localizes to ATPB‐Positive Mitochondria

**FIGURE 3:**
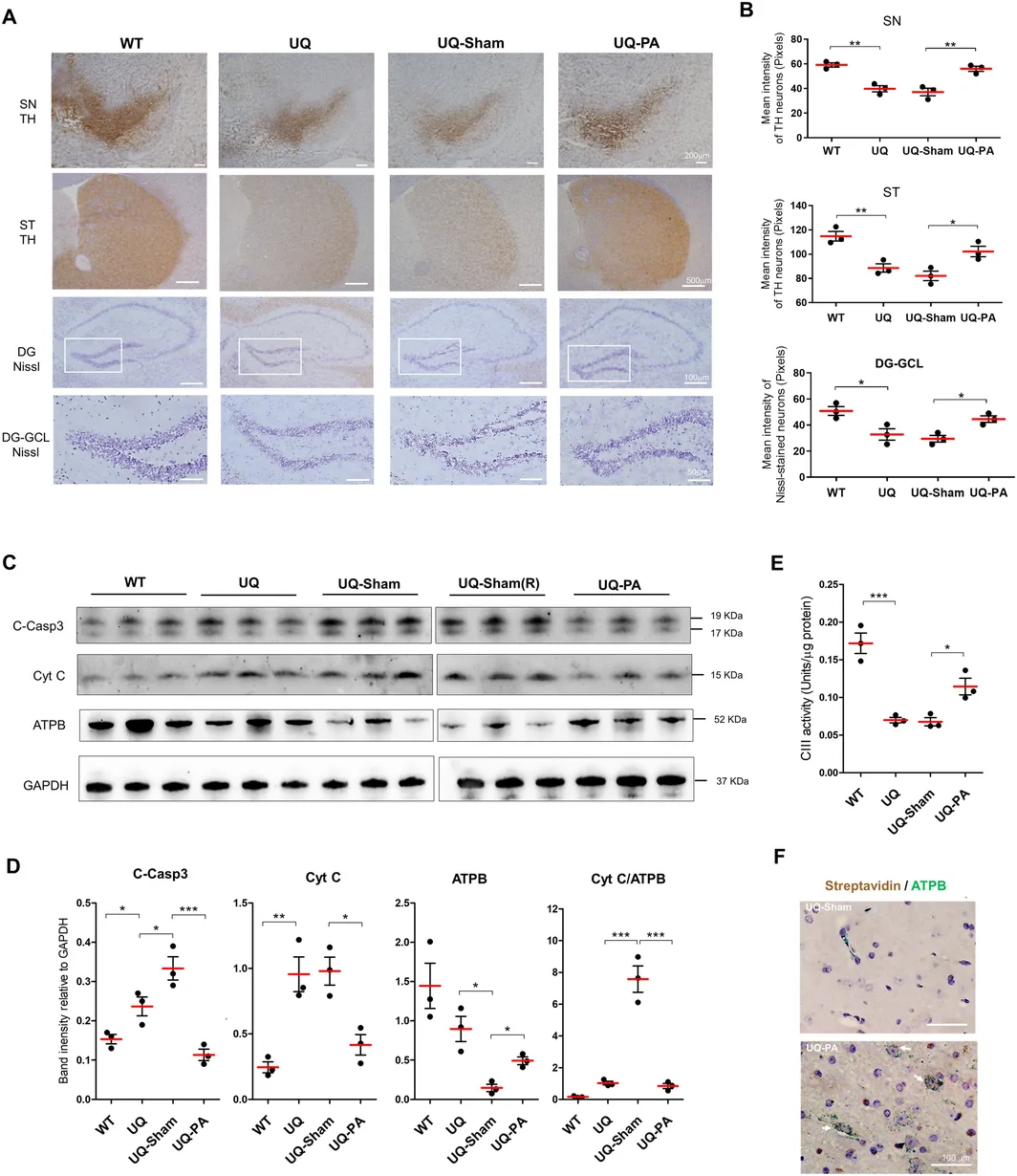
Nigrostriatal dopaminergic neuronal restoration, hippocampal structural preservation, and attenuation of mitochondrial dysfunction and apoptotic signaling in UQCRC1‐mutant mice following PenArg treatment. (A) Representative images of tyrosine hydroxylase (TH) immunohistochemistry in the substantia nigra (SN) and striatum (ST), along with Nissl staining in the dentate gyrus (DG) and granule cell layer (DG‐GCL), from Wild‐type (WT), UQCRC1‐mutant (UQ), vehicle‐treated mutant (UQ‐Sham), and PenArg‐treated mutant (UQ‐PA) mice following 6 months of intranasal administration. (B) Staining intensity of TH‐positive neurons in the SN and ST, and Nissl‐stained neurons in the DG‐GCL was quantified. (C) Representative Western blot images of cleaved caspase‐3 (C‐Casp3), cytochrome c (Cyt c), and ATP synthase subunit beta (ATPB) in the SN brain. Glyceraldehyde‐3‐phosphate dehydrogenase (GAPDH) was used as a loading control. (D) Level of C‐Casp3, Cyt c, ATPB and Cyt c/ATPB ratio were quantitated to assess the cell apoptosis and mitochondrial integrity. (E) Mitochondrial complex III (CIII) enzymatic activity in SN neurons was measured using a commercial enzymatic activity assay kit and normalized to total protein content to validate mitochondrial function. (F) Representative sagittal sections of the SN showing double immunohistochemistry with ATPB‐labeled mitochondria (green) and streptavidin‐detected PenArg (brown). In the UQ‐PA group, robust PenArg signals were observed and showed prominent colocalization with ATPB‐positive mitochondrial structures (indicated by arrows). All data are presented as mean ± SEM. *p < 0.05, **p < 0.01, ***p < 0.001; *n* = 3 per group.

TH IHC demonstrated reduced dopaminergic neuron staining in the SN and ST of UQ and UQ‐Sham mice compared with WT controls (Figure 3A,B). PenArg treatment significantly restored TH immunoreactivity in both regions. Nissl staining revealed decreased neuronal density in the DG granule cell layer (GCL) of mutant mice, which was preserved in the UQ‐PA group (Figure 3A,B). Western blot analysis of SN tissue showed increased cleaved caspase‐3 (C‐Casp3) and cytochrome c (Cyt c), together with reduced ATPB expression in mutant groups (Figure 3C,D). The Cyt c/ATPB ratio, used as an index of mitochondrial cytochrome c release relative to mitochondrial content, was elevated in UQ‐Sham mice and reduced following PenArg treatment, which also restored ATPB expression. Mitochondrial complex III (CIII) activity was decreased in mutant mice and significantly recovered in UQ‐PA mice (Figure 3E). Co‐immunohistochemistry on sagittal sections demonstrated biotin‐PenArg detection by streptavidin labeling together with ATPB staining (Figure 3F). ATPB expression (green) was increased in UQ‐PA mice, and peptide‐associated signals (brown) were observed in proximity to ATPB‐positive mitochondrial signals (arrows), whereas no detectable PenArg signal was observed in UQ‐Sham mice.

### 3.4 PenArg Distribution Across Distinct Brain Regions

**FIGURE 4:**
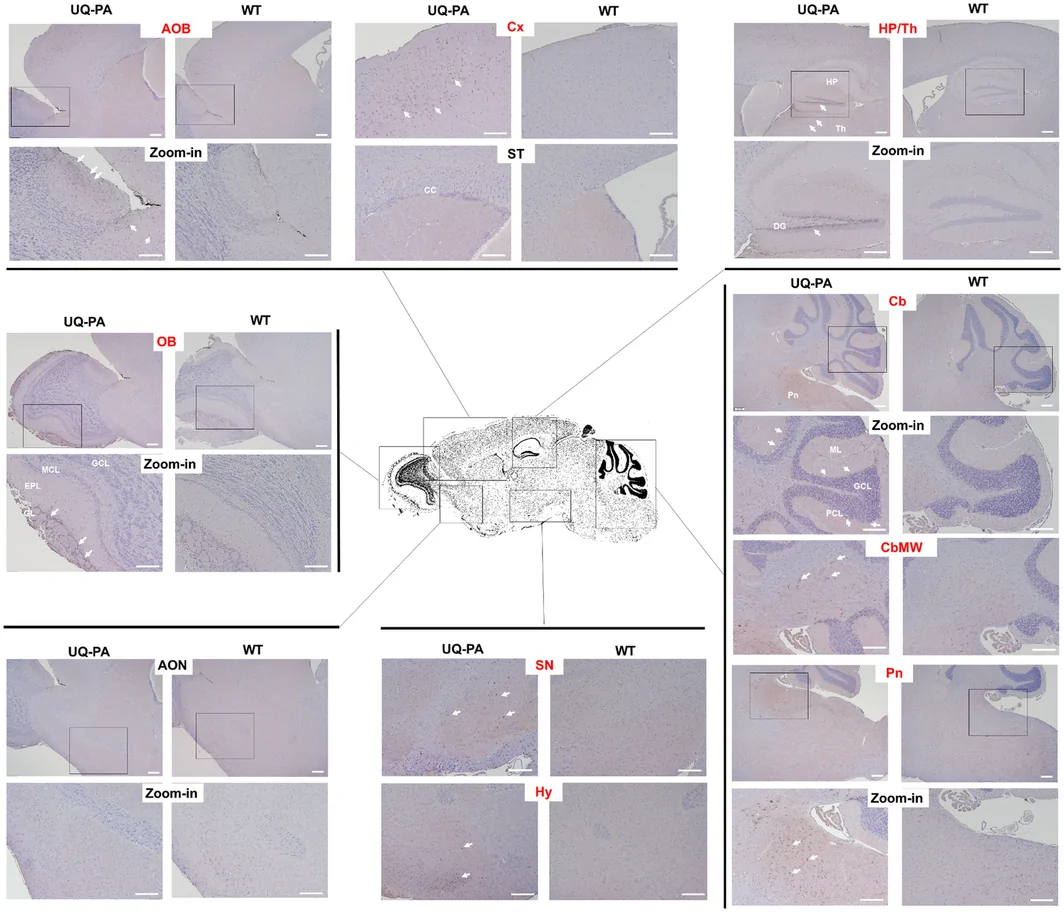
Brain‐wide distribution of PenArg after intranasal administration. Biotinylated PenArg was detected using streptavidin‐biotin immunohistochemistry after 2 h post‐intranasal administration. The central sagittal brain atlas highlighted sampled regions, with red labels indicating brain areas where PenArg‐positive signals were detected as brown chromogenic staining. Representative low‐ and high‐magnification images from PenArg‐treated and untreated wild‐type (WT) mice were shown for the anterior olfactory bulb (AOB), olfactory bulb (OB), anterior olfactory nucleus (AON), cortex (Cx), hippocampus (HP), thalamus (Th), substantia nigra (SN), hypothalamus (Hy), pons (Pn), cerebellar cortex (Cb), and cerebellar midline white matter (CbMW). Arrows indicated peptide‐positive neurons relative to WT sections. Abbreviations: Glomerular layer (GL), external plexiform layer (EPL), mitral cell layer (MCL), granule cell layer (GCL), corpus callosum (CC), molecular layer (ML), granule cell layer (GCL), Purkinje cell layer (PCL). Scale bars: 100 μm.

To assess brain distribution of intranasal PenArg, streptavidin‐biotin IHC was performed 2 h post‐administration. Figure 4 illustrates sagittal brain sections with red labels indicating regions showing PenArg‐positive brown chromogenic staining. Compared with untreated wild‐type (WT) controls, PA‐treated mice exhibited region‐specific distribution patterns, with signals detected in the anterior olfactory bulb, olfactory bulb (OB), cortex, hippocampus (Hp), thalamus, SN, hypothalamus, pons, cerebellar cortex (Cb), and cerebellar midline white matter. Untreated WT animals showed no detectable staining in corresponding regions. Higher‐magnification images (zoom‐in panels) revealed PenArg localization predominantly within neuronal layers, including the glomerular layer of the OB, the granule cell layer of the DG in the Hp, and the Purkinje cell layer of the Cb. The presence of PenArg signals in deep brain regions and brainstem structures supports rapid and effective brain penetration following intranasal administration.

### 3.5 Restoration of Skeletal Muscle Structure and Molecular Signaling

**FIGURE 5:**
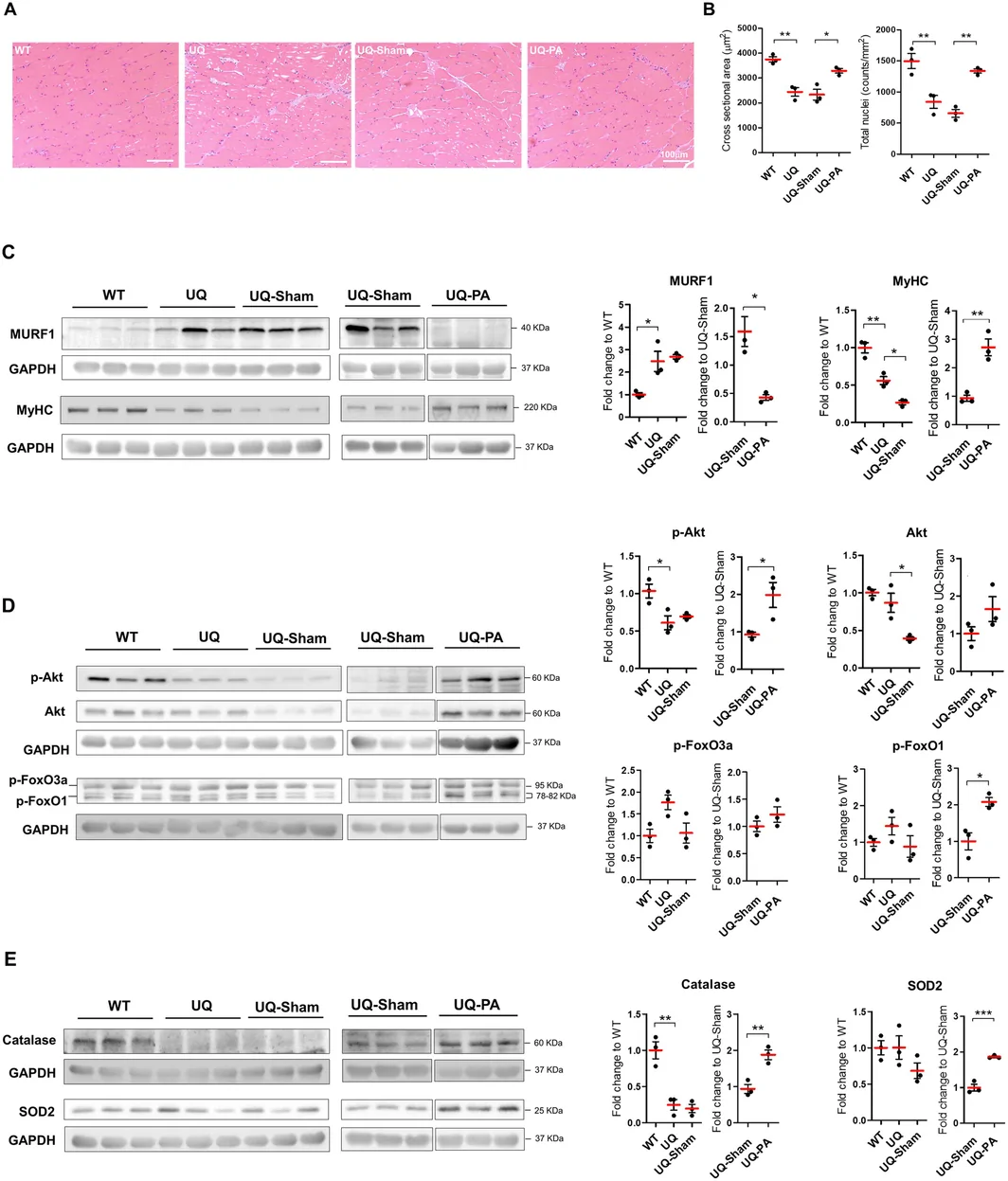
Improvement of muscle structure, anabolic signaling, and antioxidant defense in treated UQCRC1‐mutant mice. (A) Representative hematoxylin and eosin staining of tibialis anterior muscle sections from wild‐type (WT), *UQCRC1*‐mutant (UQ), vehicle‐treated mutant (UQ‐Sham), and PenArg‐treated mutant (UQ‐PA) mice. (B) Quantification of TA muscle morphology, presented as cross‐sectional area (μm^2^) and total nuclei count (nuclei/mm^2^). (C) Western blot analysis and quantification of muscle RING‐finger protein‐1 (MuRF1) and myosin heavy chain (MyHC). (D) Analysis of anabolic signaling components, including total Akt, phosphorylated Akt (p‐Akt), phosphorylated FoxO1 (p‐FoxO1), and phosphorylated FoxO3a (p‐FoxO3a). (E) Western blot analysis of antioxidant enzymes catalase and superoxide dismutase 2 (SOD2). Glyceraldehyde‐3‐phosphate dehydrogenase (GAPDH) was used as a loading control. All data are presented as mean ± SEM. Comparisons among three or more groups were performed using ordinary one‐way ANOVA followed by Tukey's multiple‐comparisons test, whereas comparisons between UQ‐Sham and UQ‐PA were performed using a two‐tailed unpaired Student's *t*‐test. *p < 0.05, **p < 0.01, ***p < 0.001, *n* = 3 per group.

After 6 months of treatment, tibialis anterior (TA) muscle from UQ and UQ‐Sham mice showed reduced fiber size and density compared with WT controls, both of which were significantly improved in UQ‐PA mice (Figure 5A,B). Western blot analysis revealed increased muscle RING‐finger protein 1 (MURF1) and reduced myosin heavy chain (MyHC) expression in mutant groups, indicating muscle atrophy, which were reversed by PenArg treatment (Figure 5C). Analysis of Akt/FoxO signaling showed reduced total Akt and phosphorylated Akt (p‐Akt) in UQ and UQ‐Sham mice, with restoration in UQ‐PA mice, particularly a significant restoration of p‐Akt (Figure 5D). Phosphorylated FoxO1 (p‐FoxO1) was significantly increased in UQ‐PA compared with UQ‐Sham, whereas phosphorylated FoxO3a (p‐FoxO3a) showed no significant differences among groups. Antioxidant analysis demonstrated reduced catalase levels in mutant mice, which were significantly restored by PenArg. SOD2 showed a similar trend without reaching statistical significance (Figure 5E).

### 3.6 Alteration of Systemic Inflammatory Cytokine Profile and Neuroinflammatory Activation in the Substantia Nigra

**FIGURE 6:**
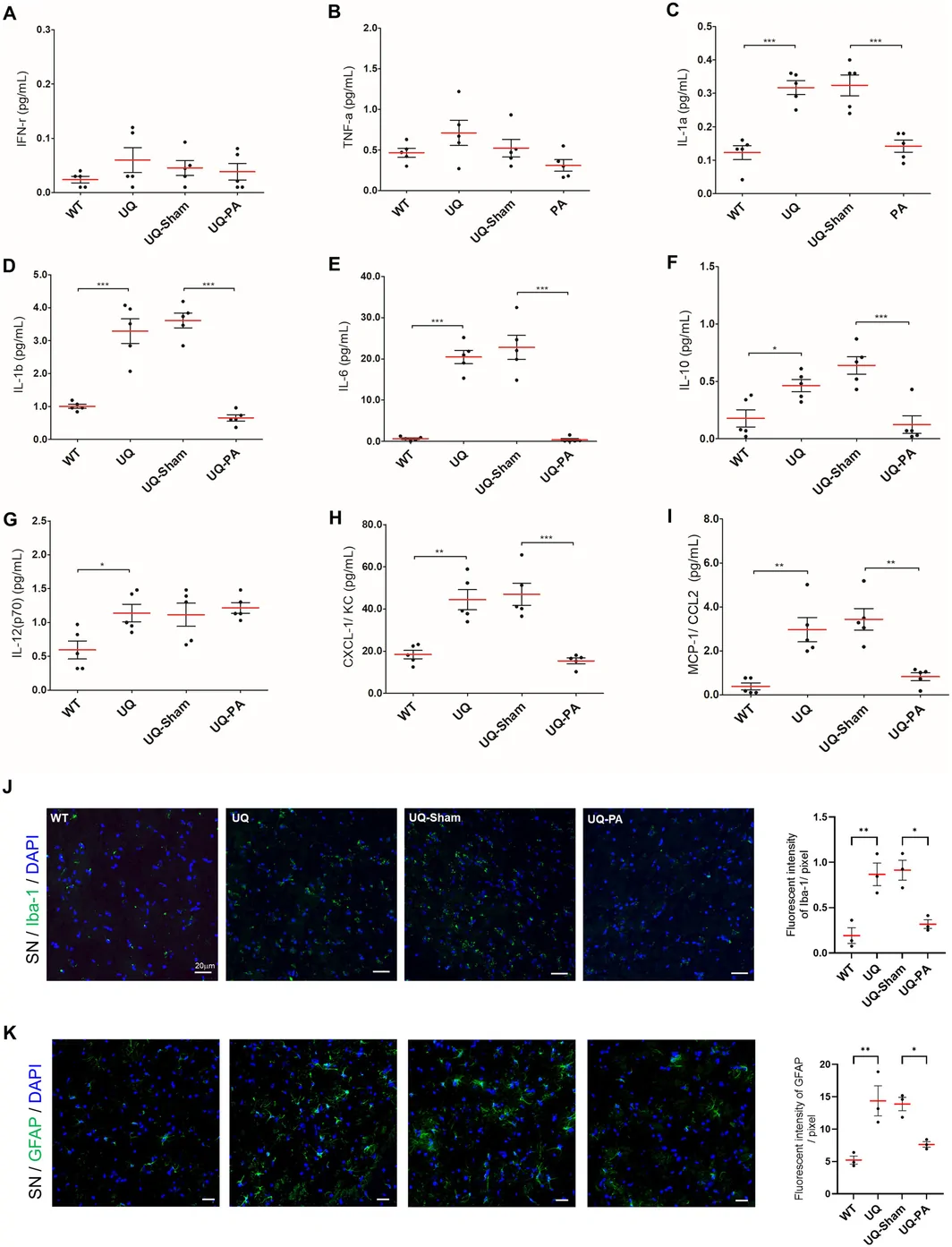
PenArg‐mediated modulation of systemic cytokine profiles in *UQCRC1*‐mutant mice. Plasma levels of inflammatory cytokines were measured using a multiplex immunoassay platform in four groups: Wild‐type (WT), UQCRC1‐mutant (UQ), vehicle‐treated mutant (UQ‐Sham), and PenArg‐treated mutant mice (UQ‐PA), after six months of intranasal administration. Cytokines analyzed included (A) interferon gamma (IFN‐γ), (B) tumor necrosis factor alpha (TNF‐α), (C) interleukin (IL)‐1α, (D) IL‐1β, (E) IL‐6, (F) IL‐10, (G) IL‐12 (p70), (H) chemokine ligand 1 (CXCL1/KC), and (I) monocyte chemoattractant protein‐1 (MCP‐1/CCL2). (J–K) Representative immunofluorescence images and quantitative analysis of Iba‐1 and GFAP expression in the substantia nigra, used as markers of microglial and astrocytic activation, respectively. All data are presented as mean ± SEM. *p < 0.05, **p < 0.01, ***p < 0.001, *n* = 5 per group.

Plasma cytokine analysis revealed no significant differences in IFN‐γ or TNF‐α among groups (Figure 6A,B). In contrast, pro‐inflammatory cytokines IL‐1α, IL‐1β, and IL‐6 were significantly elevated in UQ and UQ‐Sham mice compared with WT controls and were reduced following PenArg treatment (Figure 6C–E). CXCL1/KC and MCP‐1/CCL2 were similarly increased in mutant groups and significantly decreased in UQ‐PA mice (Figure 6H,I). IL‐12 (p70) was elevated in mutant mice but was not significantly altered by PenArg (Figure 6G). IL‐10 showed a modest increase in UQ mice and a reduction in UQ‐PA mice (Figure 6F). To confirm whether PenArg also reduced brain inflammation, Ionized calcium‐binding adapter molecule 1 (Iba‐1) and Glial fibrillary acidic protein (GFAP) immunofluorescence staining was examined in the SN. UQ and UQ‐Sham mice showed significantly increased Iba‐1 and GFAP signals compared to WT mice, indicating microglial and astrocytic activation. PenArg treatment significantly reduced both Iba‐1 and GFAP fluorescence intensity in UQ‐PA mice (Figure 6J,K). These results showed that PenArg suppresses not only systemic inflammation but also neuroinflammatory activation in the SN.

## 4 Discussion

In this study, we investigated whether PenArg, originally developed as a delivery carrier, could exert intrinsic neuroprotective effects in a genetic model of PD characterized by mitochondrial dysfunction. We demonstrate that intranasal PenArg confers neuroprotection in UQCRC1 mutation–associated PD. PenArg improved mitochondrial membrane potential and reduced apoptosis in *UQCRC1*‐mutant SH‐SY5Y cells, indicating a direct cytoprotective effect at the cellular level. Importantly, long‐term intranasal administration of PenArg ameliorated locomotor deficits and preserved dopaminergic neuron integrity in the SN and ST, as well as hippocampal structure, in mutant mice. Collectively, these findings support PenArg as a bioactive neuroprotective agent rather than merely a delivery vector [11, 20].

The *UQCRC1*‐mutant SH‐SY5Y cells showed reduced mitochondrial membrane potential and impaired filopodia formation, indicating mitochondrial dysfunction and cellular vulnerability. However, the percentage of 7‐AAD‐positive cells was not significantly increased compared with WT cells under basal conditions. This finding is consistent with an early or sublethal mitochondrial dysfunction phenotype induced by *UQCRC1* mutation, rather than overt basal cytotoxicity, because 7‐AAD positivity mainly reflects loss of plasma membrane integrity and late‐stage cell death. Under oxidative stress challenge, PenArg reduced 7‐AAD positivity and preserved mitochondrial membrane potential, supporting its protective effect against stress‐induced cell death. Specifically, low‐dose PenArg, particularly 0.5 μM, restored mitochondrial membrane potential, improved filopodia formation, and reduced oxidative stress‐induced apoptosis in *UQCRC1*‐mutant SH‐SY5Y cells. These observations align with prior reports that arginine‐rich CPPs preserve mitochondrial integrity and suppress apoptotic signaling [11, 13]. Higher concentrations attenuated protection, consistent with dose‐dependent membrane interactions reported for CPPs [21, 22, 23].

Our findings support a model in which PenArg stabilizes mitochondrial homeostasis and attenuates intrinsic apoptotic signaling, thereby preserving neuronal viability in the context of *UQCRC1*‐associated mitochondrial dysfunction. Notably, only a small fraction of PenArg localized to mitochondria, as confirmed by both in vitro and in vivo analyses. Partial mitochondrial localization is consistent with reports that guanidinium‐rich CPPs, typically composed of arginine residues, interact electrostatically with cardiolipin‐rich mitochondrial membranes [15, 17, 24, 25]. Despite partial mitochondrial localization, electrostatic interactions with cardiolipin‐rich membranes may stabilize respiratory chain activity and suppress mitochondria‐mediated apoptosis. Structurally optimized CPPs may further enhance mitochondrial targeting efficiency [26], supporting CPP‐based mitochondrial modulation strategies.

Intranasal delivery of therapeutic agents has been shown to restore mitochondrial function in PD and other CNS models [5, 18, 27], supporting the translational potential of this administration route. In the present study, PenArg was detected not only in TH‐positive dopaminergic neurons in the SN but also in the DG, where its presence was associated with preservation of hippocampal structure. These findings suggest that PenArg may exert broader neuroprotective effects beyond the nigrostriatal system. Although non‐motor outcomes were not assessed, DG preservation may be relevant to PD‐associated cognitive and affective dysfunction [28, 29]. Furthermore, given its effects on mitochondrial stabilization, oxidative stress reduction, and neuroinflammatory attenuation, PenArg may have therapeutic potential in other PD models characterized by mitochondrial dysfunction, oxidative stress, and neuroinflammation; however, further validation is required.

PenArg showed widespread brain distribution, consistent with olfactory and trigeminal nose‐to‐brain transport pathways that partially bypass the BBB [30, 31, 32], although precise mechanisms were not directly examined. In addition to central neuroprotection, PenArg conferred peripheral benefits in skeletal muscle and systemic inflammatory regulation. PenArg preserved TA muscle architecture, reduced MuRF1, restored MyHC, activated p‐Akt/p‐FoxO1 signaling, enhanced antioxidant defenses, and reduced circulating pro‐inflammatory cytokines, indicating coordinated redox–inflammatory modulation. Consistently, reduced Iba‐1 and GFAP immunofluorescence signals in the SN further support that PenArg attenuates local neuroinflammatory activation in addition to systemic inflammatory responses. Attenuation of oxidative stress may further limit downstream inflammatory signaling [33, 34], consistent with prior CPP reports [7].

Although promising, several limitations remain. PenArg showed only partial mitochondrial colocalization, and its intracellular trafficking, pharmacokinetics, and long‐term safety after intranasal delivery require further evaluation. In the present study, brain distribution was assessed only at a single time point, 2 h after intranasal administration; therefore, brain‐to‐plasma ratio, absolute nasal‐to‐brain transfer efficiency, and elimination kinetics were not determined. In addition, a positive control treatment was not included, which limits direct comparison of PenArg efficacy with established mitochondrial protective or neuroprotective agents. Representative olfactory bulb streptavidin‐biotin staining showed no overt structural disruption after three months of chronic intranasal PenArg treatment (Figure S1). However, further nasal histology and systemic toxicity studies are needed to confirm the safety of repeated intranasal PenArg administration. The potential immunogenicity of arginine‐rich CPPs in humans also warrants investigation.

## 5 Conclusion

In summary, this study demonstrates that intranasal administration of PenArg confers neuroprotection in a *UQCRC1*‐mutant model of PD by stabilizing mitochondrial homeostasis and attenuating apoptotic and inflammatory signaling. Beyond its role as a delivery vector, PenArg functions as a bioactive modulator of mitochondrial integrity, producing both central and peripheral protective effects. These findings support the therapeutic potential of CPP‐based strategies targeting mitochondrial dysfunction in neurodegenerative disorders.

## Author Contributions

Jui‐Chih Chang: conceptualization, methodology, investigation, data curation, writing – original draft preparation, writing – review and editing, project administration, funding acquisition. Cheng‐Yi Yeh: investigation, data curation, formal analysis. Kai‐Li Liu: methodology, data curation, formal analysis. Chin‐Hsien Lin: methodology, resources. Yi‐Chun Chao: data curation, formal analysis. Chin‐San Liu: supervision, funding acquisition.

## Funding

This study was supported by grants from the National Science and Technology Council (NSTC 113‐2314‐B‐371‐014) and Changhua Christian Hospital (111‐CCH‐HCR‐141).

## Ethics Statement

All animal experiments were approved by the Institutional Animal Care and Use Committee of Changhua Christian Hospital, Taiwan (approval no. CCH‐AE‐109‐011) and conducted in accordance with institutional guidelines for animal care and use.

## Conflicts of Interest

The authors declare no conflicts of interest.

## Supporting information


**Figure S1:** Representative olfactory bulb (OB) streptavidin‐biotin staining after three months of chronic intranasally biotinylated PenArg treatment. Representative OB sections from vehicle‐treated mutant (UQ‐Sham), and PenArg‐treated mutant (UQ‐PA) mice showed identifiable laminar organization, including the glomerular layer (GL), external plexiform layer (EPL), mitral cell layer (MCL), internal plexiform layer (IPL), and granule cell layer (GCL). No overt architectural disruption was observed in the UQ‐PA group compared with UQ‐Sham.

## Data Availability

The data that support the findings of this study are available from the corresponding author upon reasonable request.
