# Supplementary material for: Intranasal Administration of an Arginine‐Enriched Penetratin Peptide Confers Neuroprotection via Mitochondrial Functional Modulation in a Genetic Parkinson's Disease Model
Source: CNS Neurosci Ther. 2026 Aug 26;32(8):e71108. doi: 10.1002/cns.71108 (PMC13508580; doi:10.1002/cns.71108)
Supplement: Supplementary file 1 [file CNS-32-e71108-s001.docx]

**Supplementary results**

Representative olfactory bulb (OB) sections from UQ-Sham and UQ-PA mice are provided in Supplementary Figure 1. The major OB layers, including the glomerular layer, external plexiform layer, mitral cell layer, internal plexiform layer, and granule cell layer, were identifiable in both groups, without overt architectural disruption after three months of chronic intranasal biotinylated PA treatment. These images provide supportive downstream histological evidence but do not directly assess nasal mucosal or olfactory epithelial integrity.

Supplementary Figure 1. Representative olfactory bulb (OB) streptavidin-biotin staining after three months of chronic intranasally biotinylated PenArg treatment. Representative OB sections from vehicle-treated mutant (UQ-Sham), and PenArg-treated mutant (UQ-PA) mice showed identifiable laminar organization, including the glomerular layer (GL), external plexiform layer (EPL), mitral cell layer (MCL), internal plexiform layer (IPL), and granule cell layer (GCL). No overt architectural disruption was observed in the UQ-PA group compared with UQ-Sham.
